# A conformal Bayesian network for classification of *Mycobacterium tuberculosis* complex lineages

**DOI:** 10.1186/1471-2105-11-S3-S4

**Published:** 2010-04-29

**Authors:** Minoo Aminian, Amina Shabbeer, Kristin P Bennett

**Affiliations:** 1 Departments of Mathematical Science and Computer Science, Rensselaer Polytechnic Institute, Troy, New York, USA

## Abstract

**Background:**

We present a novel conformal Bayesian network (CBN) to classify strains of *Mycobacterium tuberculosis* Complex (MTBC) into six major genetic lineages based on two high-throuput biomarkers: mycobacterial interspersed repetitive units (MIRU) and spacer oligonucleotide typing (spoligotyping). MTBC is the causative agent of tuberculosis (TB), which remains one of the leading causes of disease and morbidity world-wide. DNA fingerprinting methods such as MIRU and spoligotyping are key components in the control and tracking of modern TB.

**Results:**

CBN is designed to exploit background knowledge about MTBC biomarkers. It can be trained on large historical TB databases of various subsets of MTBC biomarkers. During TB control efforts not all biomarkers may be available. So, CBN is designed to predict the major lineage of isolates genotyped by any combination of the PCR-based typing methods: spoligotyping and MIRU typing. CBN achieves high accuracy on three large MTBC collections consisting of over 34,737 isolates genotyped by different combinations of spoligotypes, 12 loci of MIRU, and 24 loci of MIRU. CBN captures distinct MIRU and spoligotype signatures associated with each lineage, explaining its excellent performance. Visualization of MIRU and spoligotype signatures yields insight into both how the model works and the genetic diversity of MTBC.

**Conclusions:**

CBN conforms to the available PCR-based biological markers and achieves high performance in identifying major lineages of MTBC. The method can be readily extended as new biomarkers are introduced for TB tracking and control. An online tool (http://www.cs.rpi.edu/~bennek/tbinsight/tblineage) makes the CBN model available for TB control and research efforts.

## Background

Tuberculosis (TB) is an acute or chronic infection caused by *Mycobacterium tuberculosis* complex (MTBC). TB is a leading cause of death from infectious diseases world-wide. TB genotyping enriches traditional epidemiological approaches and plays an increasingly important role in TB control strategies. It helps track transmission routes, distinguish reactivation of latent infections from potential recent transmissions, and identify outbreaks and quantify their severity. Additionally, laboratory cross-contamination events can be detected.

Multiple DNA fingerprinting methods are used for TB and their use has evolved over time. Since May 2009, two types of DNA fingerprinting methods based on PCR are routinely used for genotyping all culture-positive TB cases in the United States: spacer oligonucleotide typing (spoligotyping) and mycobacterial interspersed repetitive units – variable-number-tandem-repeat (MIRU-VNTR). Spoligotyping is based on 43 polymorphisms found in the direct repeat locus of the mycobacterial chromosome [1], while mycobacterial interspersed repetitive units (MIRU) is the number of repetitive units present in multiple loci [2].

Currently, the Centers for Disease Control and Prevention (CDC) collect spoligotype and 24 loci of MIRU for all culture positive TB patients in the US. The availability of biomarker data by each of these fingerprinting methods depends on when the method was adopted for TB control. Spoligotyping was developed first, so there are massive collections of spoligotypes maintained by the CDC and the Institute Pasteur. Subsequently, MIRU typing with 12 loci of MIRU became the standard. We refer to this set of biomarkers as 12-loci MIRU. In May 2009, spoligotyping plus 24 loci of MIRU became the standard for universal genotyping of TB in the US. We refer to this set as 24-loci MIRU. The amount of data available for each DNA fingerprinting method depends on how long that type of data has been collected. Since 2001, over forty thousand MTBC isolates have been genotyped for spoligotypes and 12-loci MIRU. A relatively small number of isolates have been typed by spoligotyping and 24-loci MIRU, since genotyping focuses primarily on current patients.

 Classification of strains of MTBC into lineages may help implement suitable control measures, especially given recent studies on the existence of stable host-pathogen associations [3] and phylogeographic distributions of strains [4]. The most definitive work for classifying strains of MTBC predominantly relies on deletion analysis to distinguish lineages [5,6]. Unfortunately, deletion analysis results are often not available in large genotyping data collections, or for routine public health TB patient investigations. So, alternatives such as mathematical models and visual rules for sublineage classification based on spoligotyping alone have been developed [7,8]. Traditionally, Restriction Fragment Length Polymorphism (RFLP) typing has also been used for lineage identification. However, this method requires maintaining live cultures of TB, which is time-consuming and the results are not comparable between labs. MIRU-VNTR*plus*[9] is a multimarker-based curated database that classifies strains by finding their nearest neighbors in the database. High accuracy was reported on classification performed using MIRU types of strains alone, which were further boosted when augmented with other biomarkers: spoligotypes, large sequence polymorphisms (LSPs), and single nucleotide polymorphisms (SNPs).

The goal of this paper is to develop a method for major lineage classification using any combination of PCR-based genotyping methods routinely collected as part of TB control and tracking efforts. When only spoligotypes are available, the model predicts the lineage using only spoligotypes. When the full set of spoligotypes plus 24- loci MIRU is available, the model predicts using all these available markers. In addition, the method should be readily adaptable to include new genomic biomarkers as they become available. The lineage classification model is trained using all available data (currently spoligotypes and up to 24 loci of MIRU), but the number of records available for each PCR-based genotype in the training set varies. At the time of prediction, the models must conform to the set of biomarkers available for prediction.

Understanding this need, this paper introduces the Conformal Bayesian Network (CBN), a probability-based model, to classify isolates into the major genetic lineages using different blends of PCR-based biomarkers. CBN identifies 6 major lineages of MTBC as identified by LSPs [4] consisting of three ancestral strains (Indo-Oceanic, *M. bovis,* and* M. africanum*) and three modern strains (Euro-American, East African Indian (CAS), and East Asian (Beijing)). Note that in East African Indian (CAS), East African Indian refers to the lineage name in [10] determined by LSPs, and CAS refers to the spoligotype family such as in [5]. This convention is also used for East Asian (Beijing). In other studies, Indo-Oceanic is also referred to by its spoligotype family name EAI, but we will not use that name here to avoid confusion.

CBN was created using two datasets provided by the CDC. The first historical dataset, *cdc1*, consists of 31482 isolates genotyped by spoligotype and 12-loci MIRU, while the second more recent dataset, *cdc2*, consists of 3255 isolated genotyped by spoligotypes and 24-loci MIRU types. Both sets comprise results from genotyping of isolates collected from TB culture-positive patients across the United States as part of TB control efforts. CBN achieves high accuracy on the CDC data and on two other independently collected datasets from MIRU-VNTR*plus* and a study in Brussels [11]. This high accuracy is maintained even when the set of DNA fingerprints used for prediction changes. The conformal model outperforms a traditional Bayesian Network constructed using only isolates genotyped by spoligotypes and 24-loci MIRU.

An online tool that classifies MTBC strains into lineages using CBN is available at http://www.cs.rpi.edu/~bennek/tbinsight/tblineage . Users may upload their strains genotyped by any combination of spoligotype, 12-loci MIRU or 24-loci MIRU. The strains are classified using CBN and the results are instantly provided.

We also visualize the probabilistic signature of spoligotype and the 24-loci MIRU profile for the CDC data. The signature extends visual rules, popularly used for spoligotypes, to MIRU, and provides insight in to the models and data.

We now provide background information on Bayesian networks, MIRU analysis, and spoligotyping.

### Bayesian network

We created a hierarchical Bayesian network (BN) to predict the 6 major lineages of the MTBC. A BN is a graphical representation of a probability distribution. Formally speaking, a BN is a directed acyclic graph  consisting of a set of nodes  to represent the variables and a set of directed links that connect pairs of nodes to represent conditional dependencies.

Each node has a conditional probability distribution that quantifies the probabilistic relation between the node and its parents, such that for a network of *k* nodes:

Therefore, one can compute the full joint probability distribution from the information in the network. In other words, a well-represented Bayesian network cancapture the complete nature of the relationship between a set of variables.

### MIRU analysis

MIRU typing based on 24 loci used in conjunction with spoligotyping has become the standard method for MTBC DNA fingerprinting in the US, allowing high-throughput, discriminatory, and reproducible analysis of clinical isolates. Because of their portable data format, spoligotypes and MIRU can potentially be used for individual strain identification based on large reference databases or classification models. Beyond studying the genetic diversity of the MTBC, MIRU has become a major method for epidemiological tracking of MTBC because of its portable data format and discriminatory power [9,12]. Altogether, there are 41 MIRU loci, of which up to 24 are used in this study. These 24 MIRU loci can be viewed as consisting of 3 subsets, MIRU locus 2677/MIRU24, MIRU1 consisting of loci 154/MIRU02, 580/MIRU04 , 960/MIRU10, 1644/MIRU16, 2059/MIRU20, 2531/MIRU23, 2996/MIRU26, 3007/MIRU27, 3192/MIRU31, 4348/MIRU39, and 802/MIRU40, and MIRU2 comprising loci 424/Mtub04, 577/ETRC, 1955/Mtub21, 2163B/QU11b, 2165/ETRA, 2347/Mtub29, 2401/Mtub30, 2461/ETRB, 3171/Mtub34, 3690/Mtub39, 4156 /QUB4156, and 4052/QUB26. We refer to MIRU locus 2677/MIRU24 by its alias MIRU24. The group 12-loci MIRU consists of MIRU1 plus locus MIRU24. The group 24-loci MIRU contains 12-loci MIRU plus MIRU2.

 	The Bayesian Network is designed to exploit the known properties of MIRU. The 24 MIRU loci are scattered throughout the chromosome of MTBC. Hence, the numbers of repeats present at each locus are independent of each other. Each locus exhibits different degrees of allelic diversity. MIRU24 is known to correspond to the TbD1 deletion, a known marker for ancestral versus modern strains [9,14]. Modern strains (i.e. Euro-American, East Asian, and East African Indian) have less than 2 repeats at locus MIRU24. With rare exceptions, ancestral strains (i.e. Indo-Oceanic, *M. bovis* and *M. Africanum*) have 2 or more repeats at MIRU24. A Hierarchical BN for major-lineage classification using MIRU has been developed [15] and forms the basis of the MIRU part of the proposed BN model.

### Spoligotyping

Spacer oligonucleotide typing (spoligotyping) is a commonly used, amplification-based method for genotyping MTBC isolates. Because the assay is inexpensive, quick, and robust, it is often used as a first-line genotyping method. It is based on the polymorphisms found in the direct repeat (DR) locus that is present in all *M. tuberculosis* complex isolates. The DR locus contains multiple 36-bp DRs separated by 30- to 40-bp unique spacer sequences [13]. Spoligotyping detects the presence or absence of 43 different spacer sequences by hybridizing labeled amplicons of the DR locus to oligonucleotide probes for each of the spacers arrayed on a membrane (a reverse line blot hybridization) [16]. Mathematically, each isolate is characterized as a 43-dimensional vector of 0s and 1s representing the presence and absence of each spacer.

A key fact about the evolution of spoligotypes is that once a spacer is lost, it is extremely unlikely to be regained. It is hypothesized that spoligotypes evolve by deletion of a single or multiple contiguous DRs, whereas insertion of DRs is very unlikely. The SPOTCLUST Bayesian Network models the asymmetric evolution of spacers using a Bayesian Network with “hidden parents” [7].The Bayesian network can be thought of as a generative model. The hidden parents of a lineage generate the members of the lineage. They capture evolution of spoligotypes without generating the full phylogeny. A spacer in the hidden parent may be lost with small probability. A spacer that is absent in the parent is almost never gained. This allows the Bayesian network to capture the deletions that are known to characterize spoligotype lineages. The hidden parent technique of SPOTCLUST is used for the spoligotype parts of the CBN model.

## Results

The following sections describe and discuss the three main results of this paper:

1) Development of the CBN model for prediction of major lineages based on available biomarkers.

2) Computational experiments establishing the effectiveness of CBN in both in- and out-of sample testing on three datasets.

3) Visualization of joint spoligotype-MIRU signatures to provide insight into TB lineages, biomarkers, and models.

### Conformal Bayesian network for mixed DNA fingerprints

**Figure 1:**
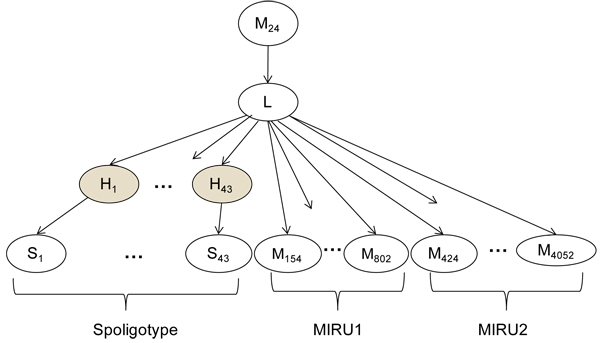
**Hierarchical Bayesian network developed to predict TB major lineages using spoligotypes and MIRU.** The model first uses *M_24_* to distinguish modern versus ancestral lineages. The spacers and MIRU are treated as conditionally independent given the lineage. The unobserved variables  capture the fact that spacers are lost and almost never gained. The shaded nodes refer to hidden variables.

      We first designed a hierarchical Bayesian Network (BN) probability model for lineage classification that captures domain knowledge about the properties of spoligotypes and MIRU. The same probability model is used by both the conformal and traditional BN. The model, shown in Figure 1, is

Where the random variable *L* represents the lineage, and the random variables  with  and  with  represent the spoligotype spacers and their hidden parents respectively. The variable *M_24_* indicates whether or not 2 or more repeats are present at MIRU24 locus. The random variables  represent the MIRU loci as indexed by their loci number.

This BN is a hierarchical generative model. The value of locus MIRU24 generates the lineage, which in turn, determines the number of repeats in the remaining MIRU loci. Thus patterns in the occurrences of repeats at each loci for each lineage are captured. The lineage also generates the hidden parents of the lineage which in turn generate the spoligotype spacers.

The BN reflects the known mechanisms of evolution of the spoligotype. As discussed above, with rare exceptions, ancestral strains have 2 or more repeats at MIRU24. Thus the top-level variable, *M_24_*, indicates whether MIRU24 is less than two (indicating modern lineages with high probability) or at least two (indicating ancestral lineages with high probability). The BN assumes that MIRU loci and the spoligotype hidden parents are conditionally independent given the lineage. The MIRU loci are scattered throughout the chromosome of MTBC in locations away from the DR locus used for spoligotypes. Thus, the assumptions of independence between the MIRU loci, and between MIRU and spoligotype, are well supported biologically. The conditional independence assumption of spacers is a model simplification previously made in the SPOTCLUST BN model [7].

Both the Conformal BN (CBN) and Traditional Bayesian Network (TBN) use the same underlying BN. The difference is in how they are trained and used for prediction. The TBN assumes there are no missing data. The training data can only contain isolates for which the spoligotypes and all 24 MIRU loci are known. To predict the lineage of a new isolate, all of the 43 spoligotypes and MIRU must be observed. In contrast, CBN is trained using all available data even if is not complete. Each conditional distribution in the model is estimated using all the data pertinent to the distribution available. The independence of the spacers and MIRU in the model makes this possible.

We have one data set (*cdc1*) consisting of 31,482 isolates genotyped by spoligotypes and 12-loci MIRU typing and one data set (*cdc2*) consisting of 3,255 isolates genotyped by spoligotypes and 24-loci MIRU typing. The CBN is trained using the information from all 34,737 isolates. The TBN can only exploit the 3,255 data points, because the original 31,482 isolates from *cdc1* are in some sense incomplete.

**Figure 2:**
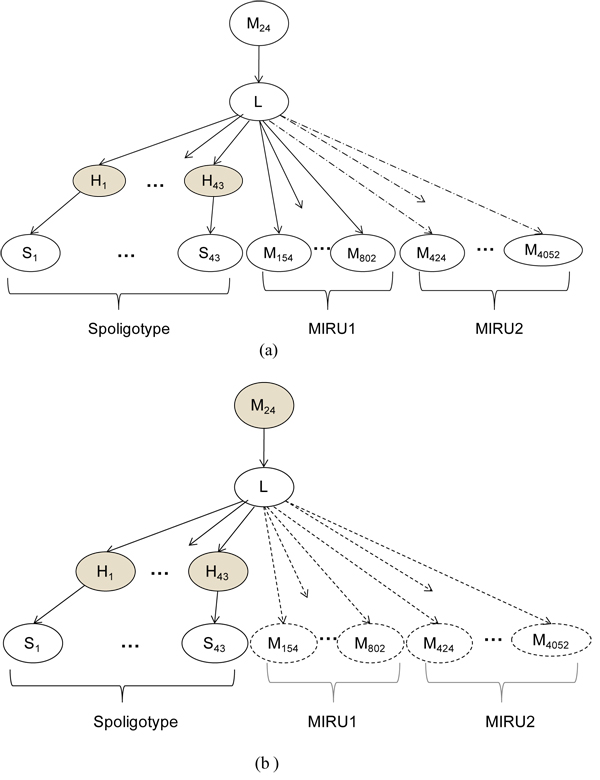
**Conformal Bayesian network (CBN) using different combinations of spoligotypes and MIRUs.** In (a) only spoligotypes and 12 loci of MIRU (MIRU1 + *M_24_*) are observed. The components of the network corresponding to the 12 loci of MIRU in MIRU2 are ignored as shown by the dotted lines. (b) CBN predicts using spoligotype only, treating *M_24_* as a missing variable and ignoring all other MIRU portions of the network. The shaded nodes refer to hidden values in each case, and the nodes represented with dotted outlines are not used for prediction.

At the time of prediction, TBN must either have all spacers and all 24 loci of MIRU available for the isolates to be predicted, or the missing biomarkers must be treated as missing values in the BN, which is a potentially expensive proposition. On the other hand, because of conditional independence of the biomarkers in the BN model, CBN can conform to the set of available biomarkers without any expensive missing value computations. None of the genotyping variables in the BN are treated as unobserved except for the hidden parent spacers (which are always unobserved) and possibly *M_24_*. Figure 2 illustrates the use of the CBN for prediction.

### Computational results

The computational experiments address three questions:

1) How well does the CBN predict the six major lineages overall?

2) Does the CBN that exploits historical data perform better than the traditional BN?

3) Can the CBN effectively predict using different subsets of the available biomarkers?

### Datasets

Four datasets were used in this study. CBN was trained using data collected by the CDC as part of routine TB surveillance in the United States from 2004-09. The CDC consists of two subsets:

(1) *cdc1*: A historical patient dataset of 31,482 isolates captures the distribution of MTBC in TB patients in the United States. It consists of spoligotypes and 12-loci MIRU.

(2) *cdc2*: A more current patient dataset of 3,255 isolates captures the distribution of MTBC in TB patients in the US. It consists of spoligotypes and 24-loci MIRU.

Two additional datasets collected and labelled in independent studies, MIRU-VNTR*plus* and Brussels [11], were used to test the models.

(3) *MIRU-VNTRplus*: A curated dataset of 163 isolates, each genotyped by spoligotype and 24-loci MIRU plus additional biomarkers. This highly curated dataset is designed to capture the genetic diversity of MTBC worldwide.

(4) *Brussels*: A patient dataset of 432 isolates reflects the distribution of strains of MTBC in patients in Brussels. The isolates are genotyped by spoligotype and 24-loci MIRU. Table 1 provides the distribution of the families within the lineages.

**Table 1:** Distribution of lineages in each dataset

	Lineage					
**Dataset**	**Total**	**Indo-Oceanic**	***M.africanum***	***M. bovis***	**Euro- American**	**East Asian**	**EastAfricanIndian**

cdc1*	31482	4409	123	583	20965	4188	1214
cdc2*	3255	531	8	78	2077	458	103
CDC	34737	4940	131	661	23042	4646	1317
Brussels	432	26	13	17	331	15	30
MIRU-VNTR*plus*	163	16	29	11	87	10	10

The datasets used in this study were (1) *cdc1* (2) *cdc2* (3) MIRU-VNTR*plus,* and (4) Brussels datasets. The number of strains of each of the 6 lineages observed is detailed in the table above. *The CDC dataset is of the union of* cdc1* and *cdc2*.

### Overall accuracy of CBN

       In the first experiment, we evaluated the overall accuracy of CBN on the CDC dataset and out-of-sample accuracy on three datasets. The model achieves excellent results overall when trained on the CDC data. Table 2 shows the confusion matrix detailing classification results on this data. The diagonal elements represent the number of strains predicted correctly for each class. Note that the total number of isolates is reported (i.e. each distinct genotype is weighted by the number of occurrences). F-values greater than 96% were reported on predictive tests on the CDC dataset for all lineages. The recall (percentage of the isolates in a given lineage correctly identified as being in that lineage) is over 99% is for all lineages. The precision (the percentage of isolates predicted to be in a lineage that are actually in the lineage) is greater than 99% for four of the six lineages. The F-value was computed as:.

**Table 2:** Overall accuracy of CBN on the CDC dataset

	Predicted Lineage						
**Recall**	**Indo-Oceanic**	***M.africanum***	***M. bovis***	**Euro-American**	**EastAsian**	**EastAfrican Indian**		

0.998	4931	7	0	1	1	0	Indo-Oceanic	
1.000	0	131	0	0	0	0	*M. africanum*	
1.000	0	0	661	0	0	0	*M. bovis*	Actual Lineage
0.994	4	3	0	22897	127	11	Euro-American	
1.000	0	0	0	0	4646	0	East Asian	
0.995	0	0	0	7	0	1310	East African Indian	
	0.999	0.929	1.000	1.000	0.973	0.992	Precision	
	0.999	0.963	1.000	0.997	0.986	0.993	F-value	

This confusion matrix for CDC dataset shows precision, recall, and F-Value for each lineage achieved by the CBN. Diagonal elements represent correctly classified cases, and off-diagonal elements indicate misclassified records.

Examining the precision and recall of the individual lineages yields further insight. The precision of East Asian is slightly lower at 97.3%. Deletion of spacers 134 is characteristic of strains of the East Asian lineage. Therefore, *P(Hj|East Asian)* ~0, for j in {1,2..34}. So, strains with many spacers missing amongst the first 34 spacers, as is the case with some Euro-American strains, are likely to be classified as East Asian. The precision for *M. africanum* drops to 92.9%, primarily because of confusion with Indo-Oceanic. This can be explained by the fact that the training set contains few strains of *M. africanum*, since strains of this lineage are rarely observed in the US. The lower recall of Euro-American (99.4%) can be explained by the existence of greater diversity in the Euro-American lineage. This is discussed further in the section about spoligotype signatures. Since no clear signature exists, strains of the Euro-American lineage get misclassified.

Finally, some of the misclassifications result because of the assumption that the MIRU24 discriminates between the ancestral and modern strains. Although the model represents this assumption based on the probability of occurrence of the number of repeats at locus MIRU24, a strain that deviates from the expected number of repeats at the MIRU24 may be misclassified.

We performed out-of-sample testing using the MIRU-VNTR*plus* and Brussels datasets [9,11] to examine the predictive accuracy of CBN. Table 3 represents the predictive accuracy as measured by F-value on these datasets. It also includes generalization results on CDC using 10% stratified cross validation of the distinct records, repeated 20 times. CBN performed well on all datasets for all lineages. The F-value on the CDC data was greater than 94.7% percent for all lineages. The F-values on the Brussels dataset were very close to the overall CDC results, with over 99% on four lineages, and slightly less accuracy on *M. africanum* and East Asian. The MIRU-VNTR*plus* dataset is designed to capture the breadth of diversity of MTBC. On that dataset Indo-Oceanic (F-value 89.7%) proved to be the most challenging, once again experiencing overlap with Euro-American.

**Table 3:** F-values of predictions made by the CBN

	F-Value				
	**Indo-Oceanic**	***M.africanum***	***M.bovis***	**Euro-American**	**EastAsian**	**EastAfricanIndian**

CDC	0.998	0.947	1.000	0.997	0.986	0.992
MIRU-VNTR*plus*	0.897	0.945	1.000	0.967	1.000	1.000
Brussels	1.000	0.917	1.000	0.994	0.938	1.000

CBN achieves high accuracy on generalization tests performed on 3 datasets (1) CDC using 10% stratified cross validation, (2) MIRU-VNTR*plus,* and (3) Brussels datasets.

### Comparison of the CBN with the Traditional Bayesian Network (TBN)

**Figure 3:**
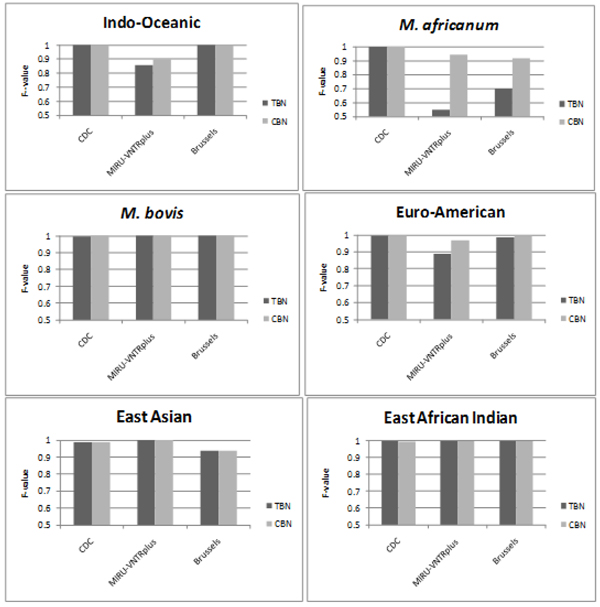
**Comparison of F-values of predictions made by the CBN and TBN for all 6 lineages.** Tests performed on 3 datasets (1) CDC using 10% stratified cross validation, (2) MIRU-VNTR*plus,* and (3) Brussels. CBN achieves equally good or better performance than TBN for all lineages on all datasets. The largest gains are seen on MIRU-VNTR*plus* and Brussels which have different distributions than the CDC dataset used for training.

      The next comparative study shows that CBN achieves better generalization than TBN by exploiting historical data even though it may be incomplete. Both TBN and CBN are trained on the CDC dataset, and then tested on MIRU-VNTR*plus* and Brussels. TBN can only be trained on the newer subset of the CDC data, *cdc2,* which has spoligotypes and all 24 loci of MIRU available, while CBN can exploit both *cdc2,* and the historical* cdc1.* To estimate the generalization on CDC, 10% stratified cross validation of the distinct records in the cdc2 dataset was repeated 20 times. TBN is trained using 90% of *cdc2*. CBN is trained using 90% of *cdc2*, plus the historical but incomplete dataset, *cdc1*, which contains only spoligotypes and 12-loci MIRU. The testing sets for TBN and CBN are identical subsets of *cdc2.* The results by lineage are shown in Figure 3. CBN provides predictions that have equal or greater accuracy than those made by the TBN, across all of the lineages, and for all of the datasets. Therefore, using all available data for training results in more powerful predictive models. For MIRU-VNTR*plus*, CBN improves generalization for Indo-Oceanic, *M. africanum,* and Euro-American. For Brussels, CBN improves generalization on *M. africanum* and Euro-American. Almost no difference exists between the performance of TBN and CBN on CDC.

The improvements occur in cases where there are differences in the underlying strain distributions in the datasets. For TBN and CBN on CDC, the training and testing sets are both drawn from *cdc2*, so adding *cdc1* to the training set of CBN does not add much more relevant information. However, the distribution of strains in cdc1, MIRU-VNTR*plus,* and Brussels are quite different. The strains of TB commonly found in patients in the US and Brussels are different. MIRU-VNTR*plus* was deliberately constructed to capture the diversity of strains worldwide, thus the underlying strain distribution is very different from both *cdc1* and *cdc2*. MIRU-VNTR*plus* includes diverse *M. africanum* strains and *M. africanum* is very rare in the US; there are only 8 *M. africanum* isolates in the *cdc2* database. The massive historical *cdc1* dataset captures more genetic diversity in these rare strains, thus it can significantly improve prediction of *M. africanum*. This experiment underscores the need for models that can exploit historical databases, even if they don’t contain all of the currently used biomarkers.

### Comparative study: use of different combinations of biomarkers

**Figure 4:**
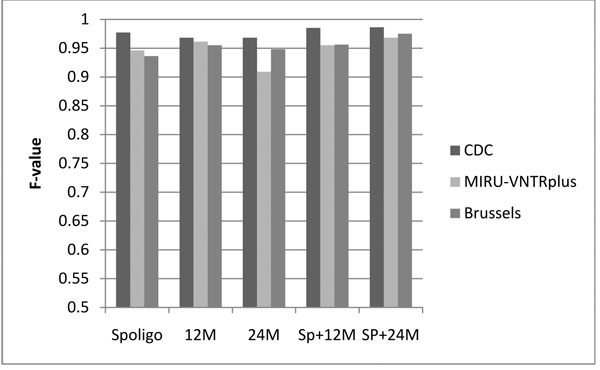
**CBN average F-value over all the lineages.** F-values obtained by CBN using different combinations of biomarkers 1) Spoligotype alone (Spoligo) 2) 12-loci MIRU (12M) 3) 24-loci MIRU (24M) 4) Spoligotype + 12-loci MIRU (Sp+12M) and 5) Spoligotype + 24-loci MIRU (Sp+24M). Out-of-sample testing was done on CDC (using 10% stratified cross-validation), MIRU-VNTR*plus* and Brussels. In general, the performance improves when the spoligotype is used in conjunction with the MIRU profile as compared to using a single type of biomarker.

**Figure 5:**
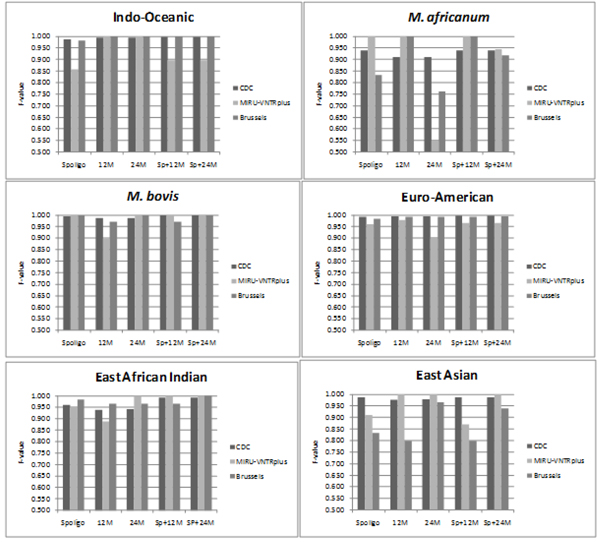
**F-values of predictions averaged over all 6 lineages.** 3 datasets were used: 1) CDC – with stratified sampling, 10% cross-validation 2) MIRU-VNTR*plus* and 3) Brussels. Results shown for all the combinations of bio-markers used: 1) Spoligotype alone (Spoligo) 2) 12-loci MIRU (12M) 3) 24-loci MIRU (24M) 4) Spoligotype + 12-loci MIRU (Sp+12M) and 5) Spoligotype + 24-loci MIRU (Sp+24M). Comparison shows that the overall performance improves when the spoligotype and MIRU are used in combination rather than individually. Improved performance is observed in most cases when 24-loci MIRU is used as compared to 12-loci MIRU.

      The next set of experiments show that CBN predicts accurately on testing data consisting of different subsets of biomarkers. Predictive tests were repeated 20 times on the CDC dataset using 10% cross-validation and the results were averaged. MIRUVNTR*plus* and Brussels were tested using a CBN model trained on the CDC data. Each test involved the use of different combinations of biomarkers for prediction (all were used for training): 1) Spoligotype alone (Spoligo), 2) 12-loci MIRU (12M) , (3) 24-loci MIRU (24M), 4) Spoligotype+12-loci MIRU (Sp+12M), and 5) Spoligotype+24-loci MIRU (Sp+24M). The overall generalization accuracy is shown in Figure 4, while the accuracy for each lineage is shown in Figure 5.

Figure 5 compares F-values obtained by CBN in out-of-sample testing using different combinations of biomarkers. In general, performance of the CBN improves or stays the same when a greater number of biomarkers are used. Improved performance is observed when spoligotype and MIRU are used in combination as compared to when they are used individually. In addition, in most cases, the F-value is higher when 24 loci of MIRU are used as compared with 12 loci. The performance of East-Asian improves considerably with the use of 24-loci MIRU, as compared to 12-loci MIRU especially on the Brussels dataset. Similar improvement in the F-values is observed on this dataset with spoligotype + 24-loci MIRU as compared to spoligotype +12-loci MIRU for the East-Asian lineage. This improvement can be explained on the basis of the marked differences between the MIRU2 profiles of East Asian and Euro-American. This is discussed further under the section on spoligotype signatures.

On the other hand for *M. africanum,* the classification accuracy is higher when 12-loci MIRU is used as compared to 24-loci MIRU. The low percentage F-value using 24 loci of MIRU can be attributed to the fact that there are very few records (8 distinct strains) of *M. africanum* for which MIRU2 data is available in the training set. Based on the performance of the model on 12-loci MIRU, if more data is available for training, the performance of the model using 24-loci MIRU can improve as well. In addition, due to the small size of the test set, the percentage recall drops greatly even if a few strains of *M. africanum* are mislabeled. Eg. In the MIRU-VNTR*plus* dataset, 3 of the 29 strains of *M. africanum* get labelled Euro-American, reducing the recall to 89.7%.

### Spoligotype and MIRU lineage signatures

**Figure 6:**
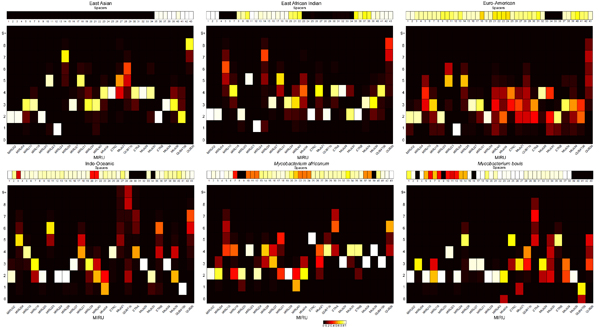
**Heat Map indicating probability distribution of spoligotype spacers and MIRU loci by lineage.** The probability of a spacer being present at each of the 43 loci of the spoligotype is shown for each lineage. Each MIRU locus is modelled as a multinomial distribution with possible values 0, 1…8, and ≥ 9 (9+). In the MIRU heat map for each lineage, the X axis represents the MIRU loci, Y axis the number of tandem repeats, and each square represents the probability of occurrence of the number of repeats at the specified locus. The range of probability values from 0 to 1 are depicted by colors ranging from black to white.

In order to construct the models we studied the probability distributions of each spacer and MIRU locus for each lineage. The visualization of these probability distributions as heat maps in Figure 6 reveals distinctive signature patterns for each lineage. We observed that the numbers of repeats at a given loci for a lineage tend to take values that lie in a close range. Distinct patterns of spoligotype spacers and MIRU loci distributions were found for each lineage. However, it is difficult to capture these patterns in simple rules or decision trees [5]. Probability-based models such as the proposed BN can do a better job of capturing the lineages than rules can. Spoligotype signatures have been previously established, in which deletions of one or more contiguous spacers have been identified as characteristic of certain lineages. [6,17]

Evidently, MIRU signatures exist too. It may be observed that, for a lineage, at a given locus, a certain number of repeats are present with high probability. The occurrence of this specific number of repeats may be unique to the lineage, and may not be observed at this locus for other lineages. Therefore, this feature may be used, possibly in combination with other markers, to identify the lineage of the strain, e.g. for *M. bovis,* 3 repeats at MTub39 and 6 repeats at ETRB are observed with high probability, while this pattern is not observed for other lineages.

One can clearly see how MIRU24 discriminates between the ancestral and modern strains with high probability. But, there are rare exceptions where MIRU24 does not discriminate between ancestral and modern strains.

An analysis of the heat map also shows that some lineages exhibit greater variability in the numbers of repeats present at MIRU loci than others. Where the color red is seen in the signatures, it indicates greater diversity in the value of the loci. This fact can be used in further dividing a lineage into its sublineages. It can be seen that for Euro-American, the number of repeats at locus QUB26 may range from 3-8, and each value occurs with an equal probability of ~0.2, as indicated by red blocks. Similar variability is observed at loci MIRU40, MTub04, MTub21, and QUB11b. The lack of a clear signature implies very few of the features occur with very low or very high probability. So, for any value of the number of repeats at a MIRU locus, there is no strong evidence of the strain belonging to the Euro-American lineage. This may explain the misclassification errors pertaining to Euro-American strains. This observation may also be viewed as evidence for the need to further classify Euro-American into sub-lineages. Definitive signatures have been established for sublineages of the Euro-American class such as Latin American Mediterranean (LAM) and Haarlem [6,17].

The greater discriminatory power of 24-loci MIRU over 12-loci MIRU combined with the spoligotype signature can help resolve the difference between some lineages. We can see that the East-Asian lineage has some very clear patterns in MIRU2. On the other hand, the Euro-American lineage shows a lot of diversity in the MIRU2 profile within the lineage. Therefore, the use of additional markers helps achieve higher classification accuracy.

An evaluation of the models and the probability distributions shows that performance may be improved by using different features. The CBN model assumes independence of each spacer in the spoligotype. But contiguous deletions characteristic of a lineage are often observed in spoligotype sequences. From Figure 6, the absence of the entire sequence of spacers from positions 1-34 is always observed for East Asian strains. Similarly, absence of spacers 39-43 is observed with high probability for strains of *M. Bovis.* Contiguous deletions may be added as variables in the CBN to account for the observance of the absence of two or more adjacent spacers. This may help solve the occasional problem of misclassification of Euro-American strains as East-Asian as observed earlier. Very rarely is a contiguous deletion from spacers 1-34 observed in Euro-American strains, while this is characteristic feature of East-Asian strains.

The number of repeats at each locus may be binned differently rather than having 10 bins for each of the numbers of repeats observed. E.g, East-Asian strains have a large number of repeats present at locus QUB26, in contrast to all other lineages. Using 2 bins, one for low and another for a high number of repeats may provide improved performance.

The study of probability distributions of biomarkers for sublineages may expose other such patterns. A detailed feature selection and evaluation process is suggested for future models that classify strains into sublineages.

## Discussion

      The existence of a broad pattern within a lineage and the significant difference in patterns across lineages observed helps explain the success of the CBN model. The structure of the hierarchical BN lends itself to creating a flexible model that can exploit a variable number of features depending upon availability. Domain knowledge such as dependence on MIRU24 to make predictions about whether a lineage is modern or ancestral, and the fact that spoligotypes are never regained once lost, are easily incorporated into the model.

 Thus, we created a simple and elegant model that incorporates domain knowledge. Classification is accomplished without having to explicitly calculate distances between genetic markers. Representing the evolutionary distances quantitatively and combining distances between different sets of biomarkers using appropriate weights can pose a challenge. Traditional distance measures fail for spoligotypes, because of the asymmetry introduced by the fact that spacers are lost but never gained. The formulation of a model for classification by alternate techniques involving distance or similarity measures, such as support vector machines may not be accomplished as effectively.

Nearest-neighbour approaches (NN) can work effectively for strain classification and can be readily used in a conformal manner. Indeed, the nearest neighbour approach used in MIRU-VNTR*plus *[9] performs well given various combinations of biomarkers. However, this approach involves selecting a suitable distance measure and cut-off. Also, changing the distance cut-off value yields varying results – a large value reduces the effect of erroneous or irrelevant values of markers. But, this results in multiple matches, possibly with different labels. In contrast, the BN determines the probability of the lineage of the strain without tuning or parameter choices based on a model that requires computational storage or time. NN algorithms require storage of the complete database.

The signature heat maps allow users to understand the decisions of the model much like prior rule-based methods based on spoligotypes [6]. Decision trees produce understandable rules that are readily interpretable. They have been used successfully for lineage classification [18], but how to incorporate TB domain knowledge, train using incomplete data, and predict using different subsets of features are all open questions in decision trees.

## Conclusions

      We have created a model using BN to accurately predict the major lineages of strains of MTBC using available PCR-based biological markers. Predictions can be made using spoligotypes, 12-loci MIRU, or 24-loci MIRU used individually or in conjunction with each other. The structure of the CBN allows it to benefit from massive historical databases which do not contain all of the biomarkers in the current standard. It can be used to predict the lineage of previously unobserved strains, even when some of the biomarkers are incomplete or unavailable.

CBN is accurate, fast, simple to train, and easy to use. It incorporates domain knowledge about spoligotypes and MIRU such as their structure, position, and mechanism of evolution. It was demonstrated that a flexible model such as the CBN is advantageous as it can exploit historical databases even though they may be incomplete. The CBN is the first probabilistic model to classify major MTBC lineages using spoligotype and MIRU. Prior BN approaches were limited to spoligotypes or MIRU alone. In this work, it was shown that, in general, the performance of the classifier improves or stays the same with an increase in the number of biomarkers used. A web-based tool for classifying major lineages based on spoligotypes and/or MIRU is available at http://www.cs.rpi.edu/~bennek/tbinsight/tblineage

Future work will involve expanding the model to predict sublineages of MTBC. The MIRU-spoligotype signatures in Figure 6 clearly show that sublineages exist within the major lineages. The exact definition of these sub-lineages is still an open question. An advantage of CBN is that can be readily used for unsupervised learning of sub-lineages based on MIRU and spoligotypes such as was done previously using a BN with spoligotypes [7]. Spoligotype signatures alone are not entirely reliable to classify strains into lineages and having the option of using additional biomarkers will help identify and analyze the specific patterns in question. In addition, we plan to explore selection of the most informative biomarkers as features of the model for each lineage while still retaining the conformal nature of the CBN model. This may further improve performance.

## Methods

### Conformal Bayesian network for efficient MTBC classification

     Details of CBN are as follows. The MIRU loci are modelled using the approach first reported in [15]. Each MIRU locus is modelled as a multinomial distribution with possible values 0, 1…8, and ≥ 9. Note all values greater than 9 are binned together since they are very rare. Since the proportions of different classes are not equal and some loci values do not occur, we use a Laplacian smoothing strategy with unequal priors. We considered the minimum probability for each value and locus pair, given the lineage, to be 0.0001. Based on this a class smoothing variable *m* was introduced and used in the following formula: For locus *i*, MIRU value *k* and lineage *L*,

where *p_i,k_* represents the overall fraction of data in lineage* L* where *M_i_* has value* k*.

 	For spoligotypes we followed the SPOTCLUST [7] model, which captures the fact that spacers are lost but almost never gained, by introducing a variable for the unobserved hidden parent (*H_j_*) and for each spacer* S_j_*, both of which follow a binomial distribution. Given a 43-dimentional spoligotype *S* and its spacer position j, let *S_j_* = 1 spacer if spacer is present, *S_j_* = 0 if spacer is absent. The probability of the spacers given the lineage is

where  and  with *H_j_* being the *j*th spacer’s hidden parent. We use *m_01_* = 10^-1^ and *m_10_* = 10^-7^. The hidden parent spacers probabilities are (except as noted below)

where  and , and *N* is the number of observations available. Note when , the correct maximum likelihood estimate is . Similarly, when , the correct maximum likelihood estimate is  When the CBN model is trained, all available data are used for every variable, so *N* would be adjusted accordingly. When the TBN model is trained, only isolates with spoligotypes and 24 loci of MIRU are used.

The CBN predicts using the subset of biomarkers available. TBN prediction is just the special case of CBN when all biomarkers are available. The probability of lineage *L* for isolate  that contains the subset of spoligotypes  and subset of MIRU loci  is:

For the case when only spoligotypes are used and MIRU24 is unknown, the lineage probability is as follows, with *m* referring to whether the strain is modern or not:

## Competing interests

The authors declare that they have no competing interests.

## Authors' contributions

KB and MA designed the model and experiments. MA performed all computational work. KB, MA and AS analyzed the results and wrote the manuscript.
